# Toxicity monitoring of solvents, hydrocarbons, and heavy metals using statistically optimized model of luminous *Vibrio* sp. 6HFE

**DOI:** 10.1186/s43141-022-00360-1

**Published:** 2022-07-01

**Authors:** Howaida Hassan, Marwa Eltarahony, Gadallah Abu-Elreesh, Hanan M. Abd-Elnaby, Soraya Sabry, Hanan Ghozlan

**Affiliations:** 1 National Institute of Oceanography and Fisheries (NIOF), Marine Environment Division, Marine Microbiology Lab., Kayet Bay, El-Anfushy, Alexandria, Egypt; 2 City of Scientific Research and Technology Applications (SRTA-City), Genetic Engineering and Biotechnology Research Institute (GEBRI), Environmental Biotechnology Department, Alexandria, Egypt; 3 Botany and Microbiology Department, Faculty of Science, Alexandria University, Alexandria, Egypt

**Keywords:** Bioluminescence, Vibrio, Sepia, Central composite design, Wastewater, Heavy metals, Hydrocarbons

## Abstract

**Background:**

The utilization of bioluminescent bacteria in environmental monitoring of water contaminates considers being a vital and powerful approach. This study aimed to isolate, optimize, and apply luminescent bacteria for toxicity monitoring of various toxicants in wastewater.

**Results:**

On the basis of light intensity, strain *Vibrio* sp. 6HFE was initially selected, physiologically/morphologically characterized, and identified using the 16SrDNA gene. The luminescence production was further optimized by employing statistical approaches (Plackett-Burman design and central composite design). The maximum bioluminescence intensity recorded 1.53 × 10^6^ CPS using optimized medium containing (g/L), yeast extract (0.2g), CaCl_2_ (4.0), MgSO_4_ (0.1), and K_2_HPO_4_ (0.1) by 2.3-fold increase within 1h. The harnessing of *Vibrio* sp. 6HFE as a bioluminescent reporter for toxicity of organic solvents was examined using a bioluminescence inhibition assay. According to IC_50_ results, the toxicity order of such pollutants was chloroform > isoamyl > acetic acid > formamide > ethyl acetate > acetonitrile > DMSO > acetone > methanol. However, among eight heavy metals tested, the bioluminescence was most sensitive to Ag^+^ and Hg^+^ and least sensitive to Co^2+^ and Ni^2+^. Additionally, the bioluminescence was inhibited by benzene, catechol, phenol, and penta-chlorophenol at 443.1, 500, 535.1, and 537.4 ppm.

**Conclusion:**

*Vibrio* sp. 6HFE succeeded in pollution detection at four different environmental and wastewater samples revealing its efficiency in ecotoxicity monitoring.

## Introduction

Water is one of the most important natural renewable resources. It is essential for the viability of all life forms. In addition, it plays a vital role in all human practices such as industrial processes, food production, agriculture, and fisheries. However, human activities have a negative impact on this precious source. Broadly, there are two sources that lead to water pollution: point and non-point sources [1]. The pollution point sources are direct certain and distinguishable sources as industries effluent, tanker release oil, or factory pipe extending into the water, while non-point sources have various numbers and different origins of contaminants that pollute both surface and groundwater such as urban wastes or runoff from agricultural fields [2]. Accordingly, water pollutants could be classified into organic and inorganic. Organohalides, herbicides, volatile organic compounds, and insecticides, etc., are the most common types of organic pollutants, whereas heavy metals, phosphates, and nitrates from industrial effluents and agriculture drainages consider being inorganic pollutants [3, 4].

Due to the water scarcity issue, it is necessary to preserve the water bodies from contamination introduced by several human anthropogenic activities. Therefore, detection of toxic organic and inorganic pollutants in environmental samples has special importance for human health and the entire ecosystem. Several literatures reported routine monitoring of water quality approaches, including chemical analysis of target toxicants and in situ assessment of indigenous biota [5].

Several limitations faced such methods; hence simple, cheap, and rapid monitoring methods were developed, especially that provide data about overall toxicity. Recently, fish, crustaceans, and algae were employed as toxicity evaluation bioassays. However, time-consuming, large sample volume, and capital intensive are the major problems associated with such organisms [6]. Therefore, the employment of microbial cells in the detection and monitoring of toxicants is advantageous in the discipline of ecotoxicological assessment and also as prober alternatives to traditional analytical methods [6].

The general idea of toxicity bioassay relies mainly on the degree of its glowing intensity either in short term or in long term by examining the change in their growth rate and viability [7]. The general mechanism for bioluminescence could be attained to the luciferase enzyme which plays a vital role in the reduction of flavin mononucleotide (FMN) to FMNH_2_, which subsequently reacts with oxygen and produce 4a-peroxy-flavin. This intermediate compound oxidizes the fatty aldehyde to a stable complex (luciferase-hydroxyflavin) which decomposes slowly and illuminates blue-green light [7, 8]. Remarkably, the first and most bioluminescent bacteria used for this purpose was *Vibrio fischeri*; it has high more sensitivity than other bacterial strains to a wide range of chemicals [7]. The bioluminescence of *V*. *fischeri* is controlled genetically through the transcription process of lux-operon (the luminescence genes). The lux-operon includes two main transcription components, luxR gene and luxICDABEG operon [7–9].

The main aim of the current study focused on the isolation, characterization, and identification of luminous bacteria and, subsequently, defining the growth medium requirements through the optimization process and ultimately applying the isolated strain in toxicity detection of various toxicants, in environmental samples from the marine aqua system and effluents.

## Materials and methods

### Invertebrate samples

Fresh different marine animals (cuttlefish, octopus, squid, and shrimp) were obtained from Maadia port, Alexandria, Egypt. Comb jellyfish was collected from the Eastern harbor of Alexandria, Egypt.

### Isolation of luminous bacteria and growth conditions

Different portions (eye, under the skin, ink sac, intestinal parts, exoskeleton, inner body, feeding tentacles, fins, and head) of collected marine invertebrates were diluted in saline (0.9%) and spread on selective luminescence agar (LA) medium of the following ingredients: 3.0 ml glycerol, 30.0g sodium chloride, 5.0g yeast extract, 5.0g peptone, 5.0, 2.5g di-potassium hydrogen sulfate, 0.25g magnesium sulfate, 1.0g calcium chloride, and 30.0 agar dissolved in 1L MiliQ water; the initial pH was 7.0 ± 0.1 [10]. The plates were incubated at 30°C for 24h [11–13]. The preliminary screening of bioluminescent colonies was performed visually in dark room. The visible luminous colony was picked and streaked on fresh LA plates for more purification. Luminous isolates were stored in 15% glycerol at −85 °C for further studies.

### Bioluminescence measurement for luminous isolates

Bioluminescence (BL) of the positive candidates was measured at regular time intervals by a luminometer (LUMISTAR, Galaxy, BMG, Germany). The intensity was measured in count per second (CPS). The measurement was done by placing 200 μl of bacterial suspension in the Greiner, Transparent, v-bottom-shaped plate. All readings were recorded in triplicate [14, 15]. The isolate which exhibited the highest emitted light was selected for subsequent identification, characterization, optimization, and application steps.

### Molecular identification of the selected luminous isolate

For identification of the selected luminescent bacterial isolate, the chromosomal DNA was extracted from a freshly prepared culture using the EZ-10 Genomic DNA kit (Bio Basic Inc., USA). The amplification of the 16S rDNA gene was performed using universal forward and reverse primers (27F; 5′AGAGTTTGATCCTGGCTCAG3 ′ and 1429 R, 5′TACGGYTACCTTGTTACGACTT3. The purified 1500-bp amplicon was subjected to sequencing by the ABI PRISM dye terminator cycle sequencing kit with Amplify Taq DNA polymerase and an Applied Biosystem (Thermo Fisher Scientific, USA). The generated sequence was submitted to the GenBank for obtaining the corresponding accession number. N-BLAST program (National Centre for Biotechnology Information) was used to analyze the sequence and compare its similarity with other sequences in the database. The software package MEGA-X was used to attain multiple alignment and phylogenetic tree.

### *Phenotypic* characterization of the selected luminous isolate

The morphological characteristics for the selected isolate were examined by a scanning electron microscope (JEOL JEM-5300, Japan). Other physiological characterestics were determined through biochemical assays by VITEK 2 Compact System, USA, and BIOMERIEUX, USA.

### Bioluminescence intensity optimization

The light intensity in fast time reaching with maintaining bioluminescence stability is the main criteria in such step. The experiment setup was conducted in a 96-well sterile tissue culture plate with 10 μl of the bacterial culture (0.5McFarland) of selected strain mixed with 190 μl of sterile broth. The optimization step in this study was carried out through two stages: one — variable at a time design (OVAT) and statistical designs including Plackett-Burman design (PBD) and central composite design (CCD).

#### One-variable-at-a-time (OVAT) approach

The maximum light intensity was examined as a function of temperature (25, 30, and 35°C), agitation (static or shaking), and incubation time. Along the experiments, the bioluminescence value (CPS) and growth (OD_600_) were recorded every hour.

#### Screening for the significant factors by PBD

PBD is a fraction of a two-level factorial design that is dedicated to screen and identify the significant variables influencing luminescence intensity emitted by the selected strain. It examines “*n*-1” variables with at least “*n*” trials. Each examined variable is represented at two levels, high (+) and low (−) [16]. According to PBD, the number of (+) is equal to (*N* + 1)/2 and the number of (−) is equal to (*N* − 1)/2 in a row. In the present study, a total of 8 (*n*) variables with two-level concentrations were studied in twelve trials as demonstrated in Table 1. The first-order model representing the Plackett-Burman experimental design is indicated by Eq. 1:

1 $$Y=\beta 0+\sum \beta iXi$$

where *Y* is the response or dependent variable (bioluminescence intensity); *β*o is the model intercept and *βi* is the linear coefficient, and *Xi* is the level of the studied variable. The significance of each factor depending on its nature (i.e., positive or negative effect on the response) was displayed by the main effect that was concluded from the statistical analysis.

**Table 1:** The studied nutritional variables with their high and low values examined in PBD

Variable	Coded values for each variable of the PBD		
	−1	0	1
**Glycerol (ml/l)**	**0.5**	**3.0**	**6.0**
**NaCl (g/l)**	**10.0**	**30.0**	**50.0**
**Yeast extract (g/l)**	**1.0**	**5.0**	**10.0**
**CaCl**_**2**_ **(g/l)**	**0.0**	**1.0**	**2.0**
**Peptone (g/l)**	**1.0**	**5.0**	**10.0**
**K**_**2**_**HPO**_**4**_ **(g/l)**	**0.5**	**2.5**	**5.0**
**MgSO**_**4**_ **(g/l)**	**0.0**	**0.25**	**0.5**
**pH**	**5**	**7**	**9**

#### Central composite design (CCD)

CCD is devoted to elucidating the interactive effect between luminescent intensity and significant variables; additionally, it predicts their optimal levels [16, 17]. Herein, yeast extract, MgSO_4_, CaCl_2_, and K_2_HPO_4_ as significant factors, determined by PBD remarkably affected luminescence intensity, were examined at 5 experimental levels: -*α*, -1, 0, +1, and + *α* in a 31-trail matrix. The concentrations of the screened significant factor at each level are illustrated in Table 2.

**Table 2:** Significant variables and their levels in CCD affecting on luminescence intensity of strain *Vibrio* sp. 6HFE

Variable	Coded levels and trial values				
	−***α***	−1	0	1	Α
**Yeast extract (g/l)**	**0.2**	**0.5**	**1**	**4**	**8**
**K**_**2**_**HPO**_**4**_ **(g/l)**	**0.1**	**0.3**	**0.5**	**2**	**4**
**MgSO**_**4**_ **(g/l)**	**0.1**	**0.3**	**0.5**	**1**	**2**
**CaCl**_**2**_ **(g/l)**	**0.5**	**1**	**2**	**3**	**4**

For statistical calculation, the relationship between the coded and actual values is represented by Eq. 2:

2 $$Xi= Ui-{Ui}_0/\varDelta Ui$$

where *Xi* is the coded value of the *i*th variable, *Ui* is the actual value of the *i*th variable, *Ui*_0_ is the actual value of the *i*th variable at the center point, and Δ*Ui* is the step change of the variable. The second-order polynomial that describes the relationship between response (luminescence intensity) (*Y*) viz the significant variables was indicated in Eq.3:

3 $$Y={\beta}_0+{\beta}_1{X}_1+{\beta}_2{X}_2+{\beta}_3{X}_3+{\beta}_{11}{X}_{11}+{\beta}_{22}{X}_{22}+{\beta}_{33}{X}_{33}+{\beta}_{12}{X}_1{X}_2+{\beta}_{13}{X}_1{\mathrm{X}}_3+{\beta}_{23}{X}_2{X}_3$$

where *Y* is the predicted response; *X*_1_, *X*_2_, and *X*_3_ are input variables which influence the response variable *Y*; *β*_0_ intercept; *β*_1_, *β*_2_, and *β*_3_ linear coefficients; *β*_11_, *β*_22_, and *β*_33_ squared or quadratic coefficients; and *β*_12_, β_13_, and *β*_23_ interaction coefficients.

### Statistical analysis

The matrices design of PBD and CCD, data interpretation, and regression modeling of the results from the experiment were statistically analyzed using Minitab 14.0 (Minitab Inc., Pennsylvania, USA), statistical software. The first analytical step for both models involves the analysis of variance (ANOVA), followed by regression analysis. Besides, the plotting profiles of CCD to predict the optimum conditions of each studied variable were also determined. The validation of the optimized model was verified through examining the predicted optimum values and comparing it with basal un-optimized media.

### Toxicity determination via bioluminescence inhibition assay (BIA)

The effect of various pollutants with different concentrations on cellular bioluminescence of the selected strain *Vibrio* sp. 6HFE was tested according to the bioluminescence inhibition assay (BIA). Initially, standard solutions of organic solvents (acetic acid, ethyl acetate, acetone, methanol, chloroform, isoamyl, acetonitrile, formamide, and DMSO), heavy metals (NiSO_4_.6H_2_O, CuCl_2_.2H_2_O, HgCl_2_, CoCl_2_.6H_2_O, AgNO_3_, FeSO_4_.7H_2_O, ZnCl_2_, and K_2_Cr_2_O_7_) and hydrocarbons (benzene, phenol, penta-chlorophenol sodium salt, and catechol) were prepared in HPLC grade sterile water. For bioluminescence assays, 100 μl of each toxicant was added to 100 μl of luminous *Vibrio* sp. 6HFE (OD = 0.4 ~ 4 × 10^6^ CFU) which was placed in a well of a microtiter plate with uniform mixing. Besides, a control was run in parallel to each toxicity well, which contained bacterial culture and sterile water. Three replicates were performed for each concentration of every toxicant. The microplate was incubated overnight in the luminometer shacked at 25°C. Bioluminescence of the culture and treated wells were measured at regular time intervals by a luminometer (LUMISTAR, Galaxy, BMG, Germany). Finally, the percent of the bioluminescence inhibition (BI %) was determined according to the following equations (4 and 5), where (*t*) is the time in min [18].

4 $$\mathrm{Bioluminescence}\kern0.5em \left(\%\right)=\frac{\mathrm{bioluminescence}\ \mathrm{of}\ \mathrm{sample}\ \mathrm{after}\kern0.5em t\kern0.5em \min }{\mathrm{bioluminescence}\ \mathrm{of}\ \mathrm{control}\ \mathrm{after}\kern0.5em t\kern0.5em \min}\times 100$$

5 $$\mathrm{Bioluminescence}\ \mathrm{inhibition}\ \left(\%\right)=100-\mathrm{Bioluminescence}\ \left(\%\right)$$

Based on BI%, the pollutant could be classified as non-toxic (0-5% BI%), possibly toxic (5-20% BI%), and toxic (> 20% BI%) as referred by [18–20]. Additionally, another parameter was taken into consideration which is IC_50_. It is the pollutant concentration which resulted in bioluminescence reduction by 50% after exposure to (*t*) time. It could be obtained from a non-linear regression relationship.

### Application of Vibrio sp. 6HFE in pollution detection at environmental water samples

Two industrial discharges (wastewater) from Tiba for food industries at the 2nd industrial Zone and AKSA Company at the 4^th^ industrial Zone, New Borg El Arab, Alexandria, in addition to two water samples from seawater of El-Dekheila and El-Max regions, Alexandria, were collected. Samples were filtered and prepared in order to be analyzed for heavy metals using Atomic Absorption spectrometer-S, Series (Thermo Fisher Scientific). For the bioluminescence assays, about 100 μl of each water sample was mixed with 100 μl of a luminous *Vibrio* sp. 6HFE culture in 96-well microtiter plates (Nunc). Consequently, the inoculated plates were subjected for luminescence detection at room temperature as previously described.

## Results and discussion

### Results

#### Isolation and identification of bacterial isolates

Judging from the light intensity of emitting luminescence, bioluminescent colonies were selected and further purified. Out of the twenty-five bacteria isolated from marine animals, only twelve showed obvious glowing with different degrees of bioluminescence as visually observed and then intensity was measured. Table 3 summarizes the visual observation, light intensity of isolates, and their isolation source.

**Table 3:** Sources of bioluminescent bacterial isolates and their bioluminescence

Isolate code	Source	Visual observation	BL intensity (CPS) × 10^**6**^
**Cr1**	**Crabs**	**+**	**1.01**
**O2**	**Octopus (inside the head)**	**++**	**1.32**
**O3**	**Octopus (inside the vitreous sac of the eyes)**	**+++**	**1.5**
**Sp4**	**Sepia (inside the vitreous sac of the eyes)**	**+++**	**1.41**
**Sp5**	**Sepia (the feeding tentacles)**	**+++**	**1.49**
**Sp6**	**Sepia (fins)**	**+**	**1.13**
**Sh7**	**Shrimp**	**++**	**1.28**
**Sq8**	**Squid (inside the vitreous sac of the eyes)**	**+++**	**1.45**
**Sq9**	**Squid (fins)**	**+**	**1.04**
**Sq10**	**Squid (dorsal)**	**+**	**1.1**
**Sq11**	**Squid (the feeding tentacles)**	**+++**	**1.42**
**Cj12**	**Comb jelly**	**++**	**1.25**

Degree of bioluminescence: strong (+++), medium (++), and weak (+)

It was observed that isolates occupying the front body part of sepia, octopus, and squid such as the head, eyes, and feeding tentacles showed more intense bioluminescence than those isolated from other body parts (Table 3).

#### Bioluminescent bacteria (BLB) identification and phylogenetic analysis

**Fig. 1:**
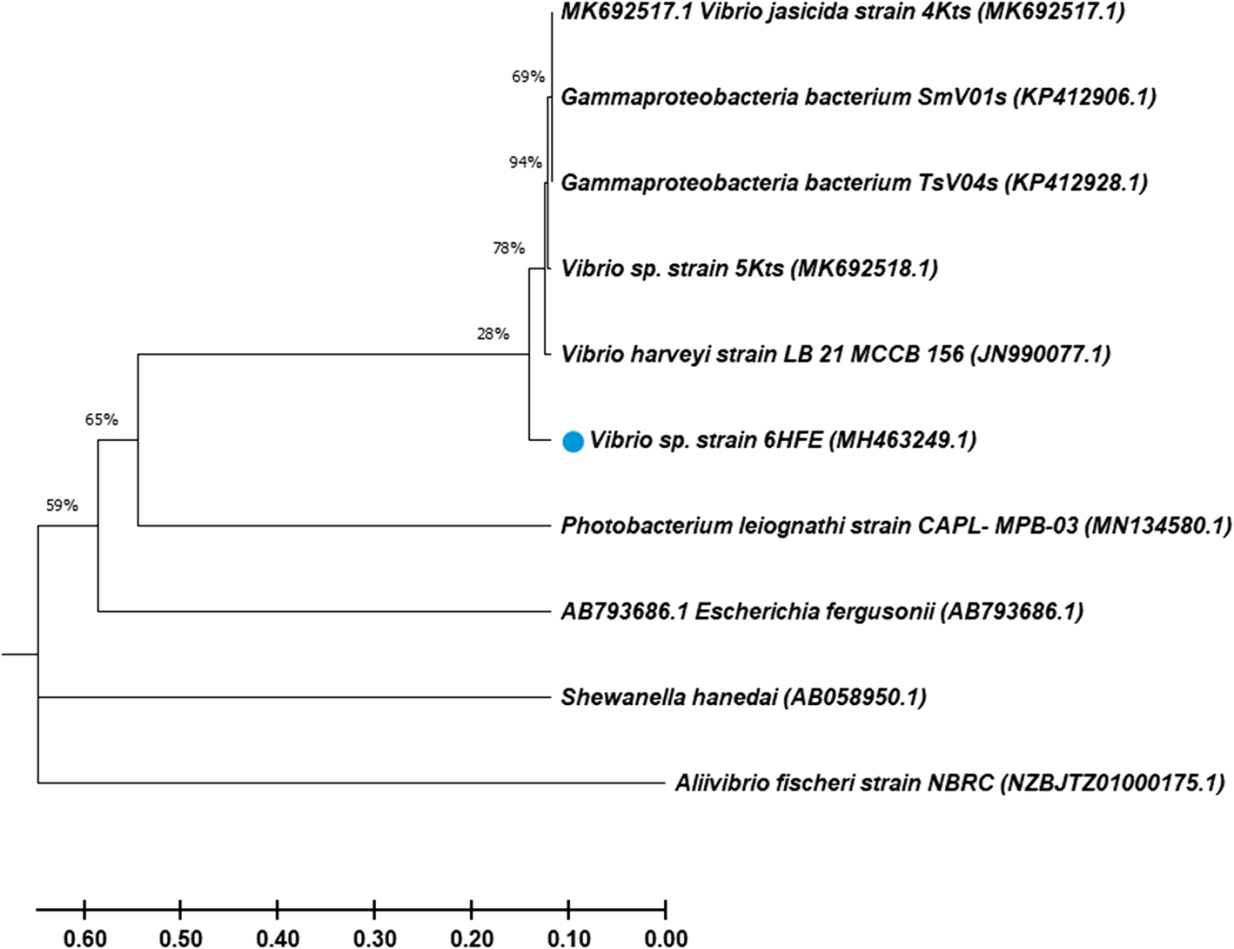
16S rDNA-based dendrogram showing the phylogenetic position of strain *Vibrio* sp. 6HFE among representative of related bacterial species

Based on the highest and the most stable intensity of blue-green light in LA media, the luminescent isolate was designated as SP5, which was chosen for further steps. The isolate was subjected to taxonomic identification via partial 16S rDNA sequences which displayed 94% DNA similarities with the number of species of the genus *Vibrio*. The sequence was deposited in GenBank with accession number MH463249 as *Vibrio* sp. 6HFE. It is affiliated to the phylum Proteobacteria, class gammaproteobacteria, order Vibrionales, and family Vibrionaceae. The phylogenetic tree of *Vibrio* sp. 6HFE was constructed by the neighbor-joining (NJ) method as indicated in Fig. 1.

#### Phenotypic and bioluminescence characterization of the selected strain

**Fig. 2:**
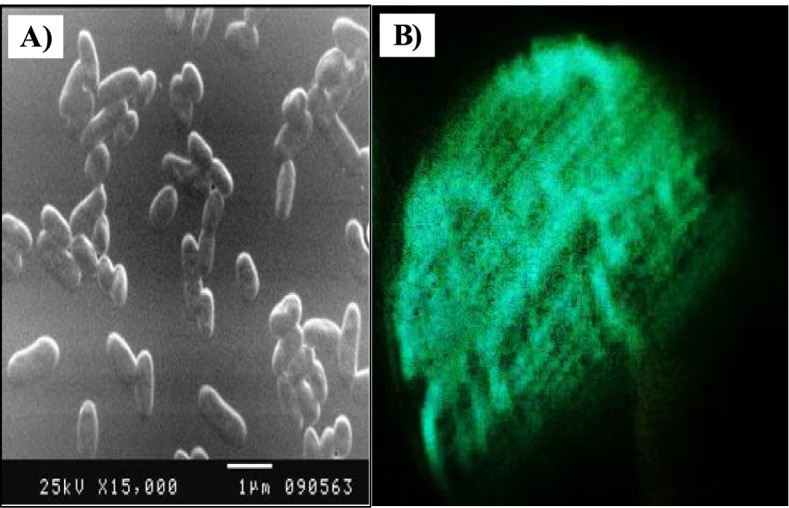
**A** SEM micrograph of *Vibrio* sp. 6EFE (×15,000); **B** bioluminescence of *Vibrio* sp. 6HFE grown on LA medium agar plates for 12 h at 30°C

*Vibrio* sp. 6EFE exhibited creamy round colonies on LA medium and turned brown as the culture becomes old. The cell morphology characteristics showed Gram-negative, with aerobic growth conditions. In addition, it appeared straight to curved non-sporulating rods with approximately 0.6 to 0.7 μm in width and 1.2 to 2.4 μm in length as shown in Fig. 2A. It grew well at the temperature range of 15-25°C, in the presence of 3-6% NaCl, and pH 7.0. For biochemical characteristics, it was positive to glucose, lactose, maltose, and methyl red tests. In addition, it was positive to indole test, gelatin hydrolysis, citrate utilization, catalase, oxidase, beta-glucosidase, L-proline arylamidase, and Glu-gly-arg-arylamidase tests and negative to urease test [21, 22]. For the bioluminescence feature, a bluish-green was emitted by *Vibrio* sp. 6EFE that was streaked on the LA plate and detected in a dark room in the absence of light as shown in Fig. 2B.

#### Bioluminescence intensity optimization

##### One variable at a time (OVAT)

**Fig. 3:**
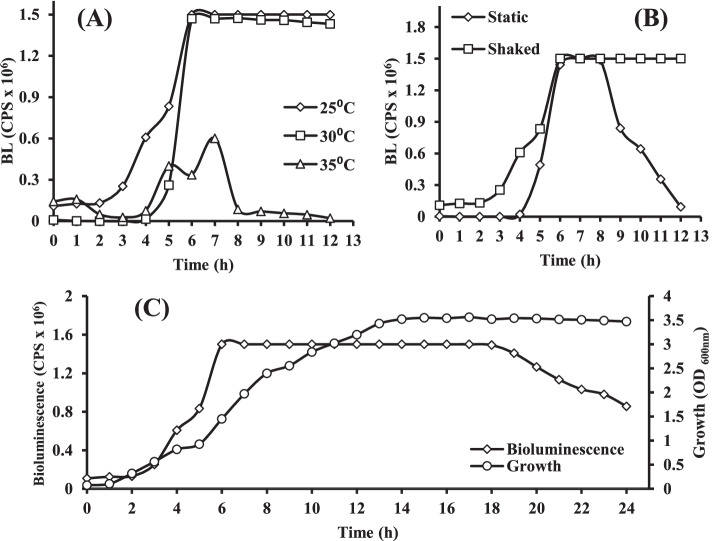
Bioluminescence of *V****ibrio*** sp. 6HFE grown on LA broth at different temperatures (**A**), under shaked and static conditions (**B**), and correlation between bioluminescence of *V****ibrio*** sp. 6HFE and its growth (**C**)

Three variables were investigated here to study their effect on the bioluminescence of *Vibrio* sp. 6HFE. The effect of temperature is illustrated in Fig. 3A. *Vibrio* sp. 6HFE displayed high-intensity values 1.5 × 10^6^ and 1.47 × 10^6^ CPS, at 25°C and 30°C, respectively, and also high stability till 6h.

At a temperature lower or higher than this range, bioluminescence decreased greatly and was inhibited completely at ˃ 30°C. For aeration effect, the bioluminescence intensity appeared higher under shaking than static condition, at 25°C for 12 h, as illustrated in Fig. 3B. The effect of incubation time is shown in Fig. 3C, and the bioluminescence intensity curve of *Vibrio* sp. 6HFE in association with the growth curve was studied at a fixed time interval over 24 h of incubation, under shaking condition at 25°C. A typical growth profile with characteristic stages (lag, logarithmic, and stationary) was displayed. In parallel, the bioluminescence intensity increased steadily till reached to its maximum value 1.5 × 10^6^ CPS at the stationary phase. However, upon 24-h incubation, the bioluminescence intensity remained stable.

##### Statistical experimental design

- Plackett-Burman design

In this study, the most significant independent parameters for maximum bioluminescence intensity were defined via PBD, which showed variation that ranged from 0.12 × 10^6^ CPS (trial number 6) to 1.39 × 10^6^ CPS (trial number 3) (Table 4). Such result disparities implied the decisive role of the optimization process in maximizing light intensity. The analysis of multiple linear regression coefficients of the model was performed by applying MINITAB 14 by Student’s *t*-test. Through using Student’s *t*-test, the error mean square was calculated in order to check the significance of the estimated coefficient of the regression equation. The variable considered as significant when its confidence level (%) is higher than 95% (prop > *F* < 0.05) [23, 24]. Thus, the most significant variables influencing the bioluminescence of *V.* sp. 6HFE were MgSO_4_, K_2_HPO_4_, CaCl_2_, and yeast extract with confidence levels of 99.6%, 97%, 97.8%, and 97.7%, respectively (Table 5).

**Table 4:** PBD matrix to evaluate the significant variables affecting BL intensity

Run order	pH	Glycerol	Yeast	Peptone	NaCl	CaCl_**2**_	MgSo_**4**_	K_**2**_HPO_**4**_	BL intensity (CPS) × 10^**6**^	
									Experimental	Predicted
**1**	**1**	**1**	**−1**	**1**	**−1**	**-1**	**−1**	**1**	**0.15**	**0.08**
**2**	**−1**	**1**	**1**	**1**	**−1**	**1**	**1**	**−1**	**1.13**	**1.08**
**3**	**1**	**−1**	**−1**	**−1**	**1**	**1**	**1**	**−1**	**1.39**	**1.31**
**4**	**−1**	**−1**	**1**	**1**	**1**	**−1**	**1**	**1**	**0.43**	**0.39**
**5**	**1**	**1**	**−1**	**1**	**1**	**−1**	**1**	**−1**	**1.02**	**1.10**
**6**	**−1**	**1**	**1**	**−1**	**1**	**−1**	**−1**	**−1**	**0.12**	**0.14**
**7**	**1**	**−1**	**1**	**1**	**−1**	**1**	**−1**	**−1**	**0.24**	**0.29**
**8**	**1**	**1**	**1**	**−1**	**1**	**1**	**−1**	**1**	**0.13**	**0.12**
**9**	**−1**	**−1**	**−1**	**1**	**1**	**1**	**−1**	**1**	**1.38**	**1.42**
**10**	**−1**	**1**	**−1**	**−1**	**−1**	**1**	**1**	**1**	**1.04**	**1.09**
**11**	**−1**	**−1**	**−1**	**−1**	**−1**	**−1**	**−1**	**−1**	**0.32**	**0.31**
**12**	**1**	**−1**	**1**	**−1**	**−1**	**−1**	**1**	**1**	**0.22**	**0.26**

**Table 5:** Statistical analysis of PBD for BL of *V*. sp. 6HFE

Term	Effect	Co-ef	SE Co-ef	***T***-value	***P***-value	Confidence level (%)
**Constant**		**465552**	**29508**	**15.78**	**0.001**	**99.9**
**pH**	**−44646**	**−22323**	**29508**	**−0.76**	**0.504**	**49.6**
**Glycerol**	**18454**	**9227**	**29508**	**0.31**	**0.775**	**22.5**
**Yeast extract**	**−254247**	**−127123**	**29508**	**−4.31**	**0.023**	**97.7**
**Peptone**	**21988**	**10994**	**29508**	**0.37**	**0.734**	**26.6**
**NaCl**	**61772**	**30886**	**29508**	**1.05**	**0.372**	**62.8**
**CaCl**_**2**_	**256794**	**128397**	**29508**	**4.35**	**0.022**	**97.8**
**MgSO**_**4**_	**479758**	**239879**	**29508**	**8.13**	**0.004**	**99.6**
**K**_**2**_**HPO**_**4**_	**−230995**	**−115497**	**29508**	**−3.91**	**0.030**	**97**
***R*-Sq = 97.58%**				***R*-Sq(adj) = 91.12%**		

**Fig. 4:**
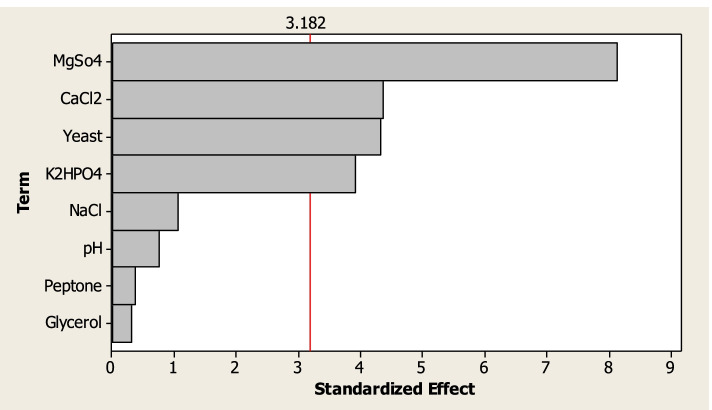
Pareto chart showed the independent variables influencing the bioluminescence of *Vibrio* sp. 6HFE

Moreover, the Pareto chart elucidated the arrangement of the examined variables affecting light intensity in descending order (Fig 4), where the vertical red line in the chart represents a *T*-value of 3.182 that indicates the least statistically significant influence for 95% confidence level. The other insignificant variables did not override the *T*-value (red line). The standard analysis of variance ANOVA of PBD clarified that the model was significant as indicated by the low probability value [*P*-value = 0.024] (Table 6). Furthermore, the overall fitting of the model was tested by evaluation of the coefficient of determination (*R*^2^) and the adjusted-*R*^2^ (adj-*R*^2^) value, which should be in reasonable agreement with the *R*^2^ value (less than 2%) [25, 26]. However, a more accurate model with higher prediction of response conjugates with an *R*^2^ value closer to 1 [24]. In our study, the model *R*^2^ and adj-*R*^2^ values recorded 0.9758 and 0.9112, respectively. Such results indicate that 97.58% of the variability of the data can be explained by the model, and there is only a 2.42% chance, which could be due to noise.

**Table 6:** ANOVA for a quadratic model of BL for *V*. sp. 6HFE

Source	DF	Seq.S.S	Adj.S.S	Adj.M.S	***F***	***P***-value
**Main effects**	**8**	**1.26223E+12**	**1.26223E+12**	**1.57779E+11**	**15.10**	**0.024**
**Residual error**	**3**	**31345296192**	**31345296192**	**10448432064**		
**Total**	**11**	**1.29358E+12**				

The first-order model for bioluminescence intensity obtained by ANOVA was fitted to the results obtained from the 12-experiment matrix, which indicated the response (bioluminescence intensity) as a function of eight studied parameters (Eq. 6).

6 $${\displaystyle \begin{array}{l}Y\left(\mathrm{BL}\right)=465552-22323\ \mathrm{pH}+9227\ \mathrm{glycerol}-127123\ \mathrm{yeast}+10994\ \mathrm{peptone}\\ {}+30886\ \mathrm{NaCl}+128397\ {\mathrm{CaCl}}_2+\kern0.5em 239879\ {\mathrm{MgSO}}_4-115497\ {\mathrm{K}}_2{\mathrm{HPO}}_4\end{array}}$$

For the subsequent stage of optimization (central composite design CCD), all parameters with a positive influence on response were remained constant at their high level, and those parameters negatively influenced were fixed at their low level.

- Central composition design (CCD)

To obtain the optimum response (bioluminescence intensity), CCD is an ideal technique that was used to predict exactly the most effective concentration values of the significant variables [27]. It consisted of five levels (−*α*, −1, 0, +1, + *α*) with 4 independent variables which were yeast extract, MgSO_4_, K_2_HPO_4_, and CaCl_2_ for *Vibrio* sp. 6HFE. About 31 trials were performed with their response, and they included 7 replicates of central points, 16 factorial (cubic points), and 8 axial (star point) as the risk of missing non-linear relationships has to be minimized in the middle of the intervals. The repetition is important to calculate the confidence intervals [28]. Table 7 represents different combinations of examined significant parameters in the matrix along with the actual and predicted response. As illustrated, bioluminescence intensity varied among the examined trials displaying maximum response with 1.50 × 10^6^ CPS at trial 16 (axial point) and minimum response with 0.02 × 10^6^ CPS at trial 14 (factorial point).

- Statistical analysis of the data

**Table 7:** CCD matrix of variables influencing V. sp. 6HFE BL intensity

Run	Yeast	MgSO_**4**_	CaCl_**2**_	K_**2**_HPO_**4**_	BL intensity (CPS) × 10^**6**^	
					Experimental	Predicted
**1**	**0**	**0**	**0**	**0**	**1.45**	**1.36**
**2**	**−1**	**1**	**−1**	**1**	**0.30**	**0.42**
**3**	**1**	**1**	**1**	**1**	**0.62**	**0.77**
**4**	**0**	**2**	**0**	**0**	**1.25**	**1.09**
**5**	**0**	**0**	**0**	**0**	**1.18**	**1.36**
**6**	**1**	**−1**	**1**	**−1**	**0.59**	**0.49**
**7**	**1**	**1**	**−1**	**−1**	**0.99**	**1.13**
**8**	**−1**	**−1**	**−1**	**1**	**1.30**	**1.25**
**9**	**1**	**1**	**1**	**−1**	**0.79**	**0.90**
**10**	**−2**	**0**	**0**	**0**	**0.14**	**0.27**
**11**	**1**	**−1**	**−1**	**1**	**0.04**	**0.21**
**12**	**−1**	**1**	**1**	**−1**	**0.38**	**0.23**
**13**	**−1**	**−1**	**1**	**−1**	**1.46**	**1.55**
**14**	**−1**	**1**	**1**	**1**	**0.02**	**0.09**
**15**	**0**	**0**	**0**	**0**	**1.15**	**1.36**
**16**	**0**	**0**	**−2**	**0**	**1.50**	**1.44**
**17**	**−1**	**−1**	**1**	**1**	**1.49**	**1.37**
**18**	**0**	**0**	**0**	**0**	**1.49**	**1.36**
**19**	**1**	**−1**	**1**	**1**	**0.25**	**0.32**
**20**	**0**	**0**	**0**	**2**	**0.25**	**0.10**
**21**	**0**	**0**	**0**	**0**	**1.36**	**1.36**
**22**	**1**	**−1**	**−1**	**−1**	**0.28**	**0.26**
**23**	**−1**	**−1**	**−1**	**−1**	**1.43**	**1.31**
**24**	**0**	**0**	**0**	**0**	**1.39**	**1.36**
**25**	**0**	**0**	**2**	**0**	**1.35**	**1.33**
**26**	**0**	**0**	**0**	**−2**	**0.21**	**0.29**
**27**	**0**	**0**	**0**	**0**	**1.49**	**1.36**
**28**	**1**	**1**	**−1**	**1**	**1.14**	**1.11**
**29**	**2**	**0**	**0**	**0**	**0.11**	**0.10**
**30**	**0**	**−2**	**0**	**0**	**1.42**	**1.50**
**31**	**−1**	**1**	**−1**	**−1**	**0.46**	**0.45**

Table 8 indicates the multiple regression analysis of the data, coefficients, *P-*value, and *F*-value. The model determination coefficient (*R*^2^) of 95.3% referred to the goodness of fit for the model (Table 8). The adjusted *R*^2^ value recorded 91.2% pointing out to the high efficiency and significance. Besides, data were judged based on the significance (*P* < 0.05).

**Table 8:** Determination regression coefficient of second-order polynomial model for *Vibrio* sp. 6HFE bioluminescence

Term	Coef.	SE Coef.	***T***-value	***P***-value
**Constant**	1356954	62059	21.866	**0.000**
**Yeast extract**	−92777	33516	−2.768	**0.014**
**MgSO**_**4**_	−102822	33516	−3.068	**0.007**
**CaCl**_**2**_	−26553	33516	−0.792	**0.440**
**K**_**2**_**HPO**_**4**_	−47855	33516	−1.428	**0.173**
Yeast extract* yeast extract	−317865	30705	−10.352	**0.000**
**MgSO**_4_*MgSO_**4**_	−15062	30705	−0.491	**0.630**
**CaCl**_2_*CaCl_**2**_	7473	30705	0.243	**0.811**
**K**_**2**_**HPO**_4_*K_**2**_**HPO**_**4**_	−291506	30705	−9.494	**0.000**
Yeast extract *MgSO_**4**_	431849	41048	10.521	**0.000**
Yeast extract *CaCl_**2**_	−4030	41048	−0.098	**0.923**
Yeast extract *K_**2**_**HPO**_**4**_	1241	41048	0.030	**0.976**
**MgSO**_4_*CaCl_**2**_	−114325	41048	−2.785	**0.013**
**MgSO**_4_*K_**2**_**HPO**_**4**_	10236	41048	0.249	**0.806**
**CaCl**_2_*K_**2**_**HPO**_**4**_	−28726	41048	−.700	**0.494**

***R*-Sq = 95.3%** ***R*-Sq(adj) = 91.2%**

Statistical analysis of the data manifested that the model is significant, as demonstrated by a very low *P*-value (0.00 < 0.05) (Table 9). It is clear from the significance values that the linear coefficients of yeast extract, MgSO_4_, and quadratic effects of yeast extract and K_2_HPO_4_ are significant (Table 8). The *P-*values pointed out that the linear coefficient of CaCl_2_ and K_2_HPO_4_ was not significant (*P-*value = 0.440 and 0.173, respectively), whereas the majority of interactions between the studied variables were not significant, except yeast extract and MgSO_4_ and CaCl_2_ and MgSO_4_ (*P*-values of 0.00 and 0.013, respectively), implying that they did not contribute significantly to the maximization of response. However, an additional factor that considers being a determinant for estimating the quality of the model is the lack of fit. It characterizes the variation in the data around the fitted design testified.

**Table 9:** ANOVA of the quadratic polynomial model for BL of *Vibrio* sp. 6HFE

Source	DF	Seq SS	Adj SS	Adj MS	F	P
**Regression**	**14**	**8.71958E+12**	**8.71958E+12**	**6.22827E+11**	**23.10**	**0.000**
**Linear**	**4**	**5.32199E+11**	**5.32199E+11**	**1.33050E+11**	**4.94**	**0.009**
**Square**	**4**	**4.97921E+12**	**4.97921E+12**	**1.24480E+12**	**46.17**	**0.000**
**Interaction**	**6**	**3.20818E+12**	**3.20818E+12**	**5.34696E+11**	**19.83**	**0.000**
**Residual error**	**16**	**4.31349E+11**	**4.31349E+11**	**26959290759**		
**Lack-of-fit**	**10**	**3.10610E+11**	**3.10610E+11**	**31060968406**	**1.54**	**0.308**
**Pure error**	**6**	**1.20739E+11**	**1.20739E+11**	**20123161346**		
**Total**	**30**	**9.15093E+12**				

The insignificant lack of fit ensures a good design; it indicated that there might be contributions in the regresses-response relationship, which are not considered by the design [29]. Insignificant lack of fit is required, and herein, it recorded 0.308. To evaluate the correlation between the tested four parameters and to calculate the highest light intensity corresponding to the optimal values of yeast extract, CaCl_2,_ MgSO_4_, and K_2_HPO_4_, the equation of the second-order polynomial model (Eq. 7) has been suggested. Based on the optimum levels of the independent parameters, the maximum bioluminescence intensity was predictable:

7 $${\displaystyle \begin{array}{l}\mathbf{Y}\left(\mathbf{BL}\right)=1356954-92777\kern0.5em \mathbf{yeast}\ \mathbf{extract}-102822\kern0.5em {\mathbf{MgSO}}_{\mathbf{4}}-26553\kern0.5em {\mathbf{CaCl}}_{\mathbf{2}}-47855\kern0.5em {\mathbf{K}}_{\mathbf{2}}{\mathbf{HPO}}_{\mathbf{4}}\\ {}-317865\kern0.5em \mathbf{yeast}\ \mathbf{extract}\ast \mathbf{yeast}\ \mathbf{extract}-15062{\mathbf{MgSO}}_{\mathbf{4}}\ast {\mathbf{MgSO}}_{\mathbf{4}}+7473{\mathbf{CaCl}}_{\mathbf{2}}\ast \\ {}{\mathbf{CaCl}}_{\mathbf{2}}-291506\kern0.5em {\mathbf{K}}_{\mathbf{2}}{\mathbf{HPO}}_{\mathbf{4}}\ast {\mathbf{K}}_{\mathbf{2}}{\mathbf{HPO}}_{\mathbf{4}}+431849\kern0.5em \mathrm{yeast}\ \mathrm{extract}\ast {\mathrm{MgSO}}_4-\\ {}4030\kern0.5em \mathbf{yeast}\ \mathbf{extract}\ast {\mathbf{CaCl}}_{\mathbf{2}}+1241\kern0.5em \mathrm{yeast}\ \mathrm{extract}\ast {\mathrm{K}}_2{\mathrm{HPO}}_4-114325\kern0.5em {\mathbf{MgSO}}_{\mathbf{4}}\\ {}\ast {\mathbf{CaCl}}_{\mathbf{2}}+10236\kern0.5em {\mathbf{MgSO}}_{\mathbf{4}}\ast {\mathbf{K}}_{\mathbf{2}}{\mathbf{HPO}}_{\mathbf{4}}-28726{\mathbf{CaCl}}_{\mathbf{2}}\ast {\mathbf{K}}_{\mathbf{2}}{\mathbf{HPO}}_{\mathbf{4}}\end{array}}$$

**Fig. 5:**
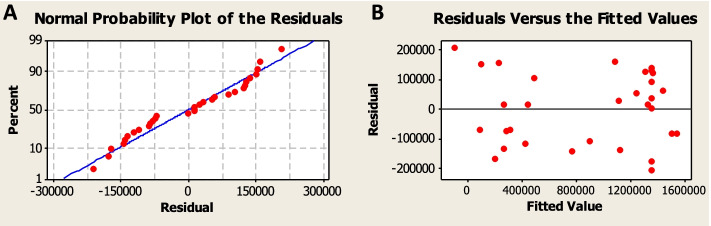
**A** Normal probability plot of residuals against bioluminescence, **B** residual distribution against fitted values plot of RSM

For checking the accuracy of the model, normal probability plot and residual plots were evaluated. The normal probability plot is a useful graphical method to characterize the nature of the residuals and validate models (Fig. 5). All residuals are close to the diagonal line in a way that indicates they are normally distributed. In the residual plots, all residuals are randomly and uniformly scattered against the fitted values of the model. Therefore, all previous evidence reflects a good model fit and affirms the model’s adequacy.

- Graphical plots for interaction effect representation

**Fig. 6:**
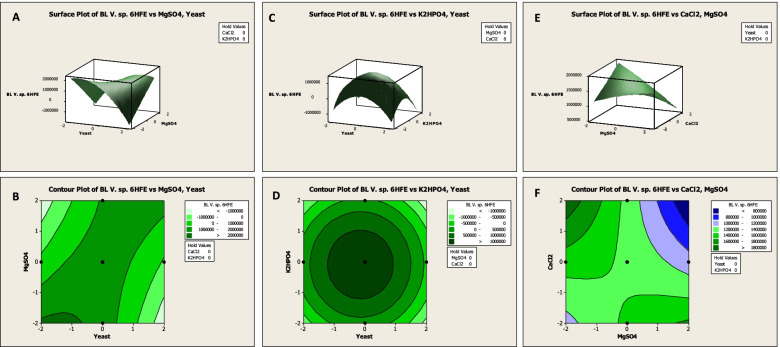
3D surface plot (left panels) and 2D contour plot (right panels) displaying the mutual effects of independent significant variables on bioluminescence intensity

The three-dimensional surface and two-dimensional contour plots were designed to illustrate the interaction effect between response (bioluminescence intensity) and examined independent parameters, besides identifying and predicting the optimal concentrations for maximal response. As observed in Fig. 6A, bioluminescence intensity was plotted on the *Z*-axis against yeast extract and MgSO_4_, while the other factors were set at their zero level. It showed that when concentrations of both variables decreased, the bioluminescence intensity gradually increased. The saddle contour plot (Fig. 6B) reflected the significant synergistic interaction between them. Generally, the shape of the contour plot reveals the nature and extent of the interactions between the variables. However, a circular contour plot highlights an insignificant interaction between variables [27, 30]. Consequently, the correlation between yeast extract and K_2_HPO_4_ could be described as insignificant, as indicated by the circular 2D-contour plot (Fig. 6D). Furthermore, at the moderate level of both factors, the highest response is obtained in Fig. 6C. However, the antagonistic correlation could describe the interaction effect of MgSO_4_ and CaCl_2_ Fig. 6E, where the maximum bioluminescence intensity could be achieved by increasing the concentration of CaCl_2_ and decreasing MgSO_4_ concentration or vice versa (Fig. 6F).

**Fig. 7:**
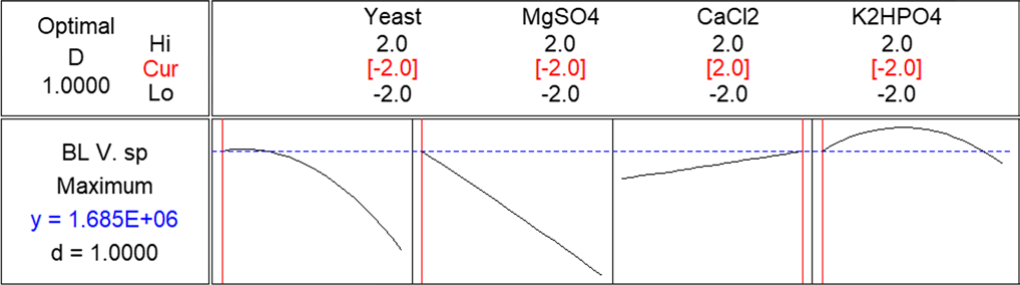
Response optimizer and desirability function of *V.* sp. 6HFE bioluminescence

To attain maximum bioluminescence, the response optimizer tool in MINITAB 14 was applied by solving the reduced regression models. This tool calculates the individual desirability using a transfer or desirability function. As the composite desirability reached its maximum, the optimal response was obtained. Always, the response optimizer is between zero and one. When it equals one, it means that the response (bioluminescence intensity) becomes at the ideal case. But when it equals zero, it indicates that the response is outside agreeable limits. The response optimizer at optimum conditions for maximum targets is shown in Fig. 7.

The desirability value for *V*. sp. 6HFE is 1.0, confirming the fitting of the performed design that results in maximum bioluminescence (1.5 × 10^6^ CPS). The optimum predicted concentrations (g/L) of the significant variables for *V.* sp. 6HFE were yeast extract, 0.2; CaCl_2_, 4.0; MgSO_4_, 0.1; and K_2_HPO_4_, 0.1.

- Verification of the model

The validation of the experimental results of *V.* sp. 6HFE for bioluminescence intensity was evaluated by comparing optimum conditions predicted from CCD and basal un-optimized conditions. This optimization strategy succeeded in maximizing bioluminescence intensity within 1 h from (initial basal) 0.66 × 10^6^ CPS to 1.5 × 10^6^ CPS (predicted from CCD) by a 2.3-fold increase.

##### Application of *Vibrio* sp. 6HFE as biosensor

- BIA for various solvents

**Fig. 8.**
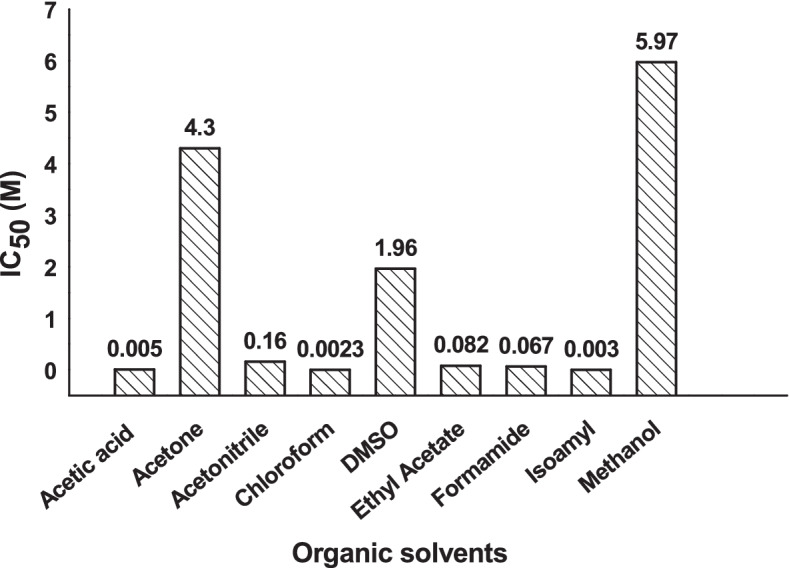
IC_50_ of organic solvents on the bioluminescence of *Vibrio* sp. 6HFE

The toxicity effect of nine solvents with different categories, polarities, and different concentrations ranging from 0.006 to 12.3 M was tested on the bioluminescence of *Vibrio* sp. 6HFE for 5 min, the selected shortest exposure time bioassay. Based on IC_50_ results of all examined solvents (Fig. 8), the toxicity of the nine organic solvents could be arranged from the most toxic to the least toxic as the following: chloroform > isoamyl > acetic acid > formamide > ethyl acetate > acetonitrile > DMSO > acetone > methanol.

- BIA for heavy metals﻿

**Fig. 9:**
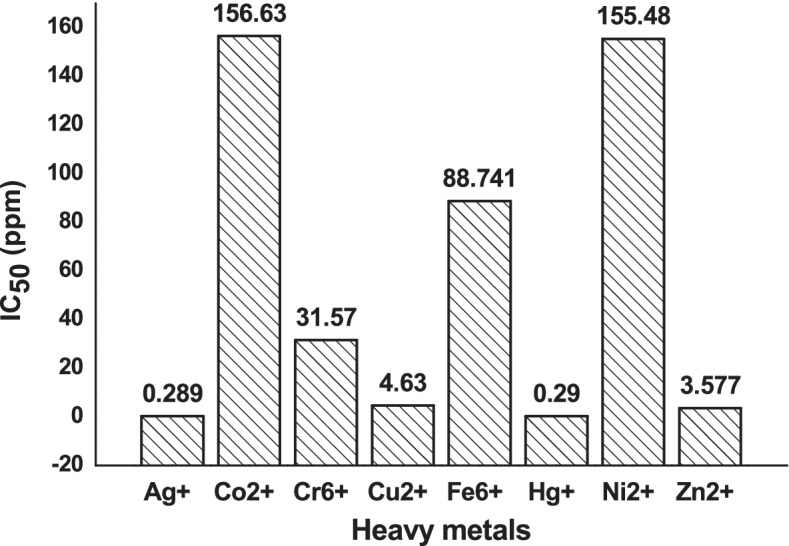
IC_50_ of heavy metals on the bioluminescence of *Vibrio* sp. 6HFE

The bioluminescence profile of *V.* sp. 6HFE in the presence of eight heavy metals was studied (Cr^6+^, Co^2+^, Cu^2+^, Fe^2+^, Hg^+^, Ag^+^, Ni^2+^, and Zn^2+^) at concentrations (1 to 1000 ppm). Obviously, silver and mercury completely inhibited the bioluminescence in all examined concentrations. The IC_50_ values of examined heavy metals for the bioluminescence of *Vibrio* sp. 6HFE are represented in Fig. 9. Accordingly, the order of toxicity for bioluminescence of *Vibrio* sp. 6HFE could be expressed from the most toxic to the least toxic as the following: Ag^+^ > Hg^+^ > Zn^2+^ > Cu^2+^ > Cr^6+^ > Fe^6+^ > Co^2+^ > Ni^2+^.

- BIA for aromatic hydrocarbon compounds﻿

**Fig. 10:**
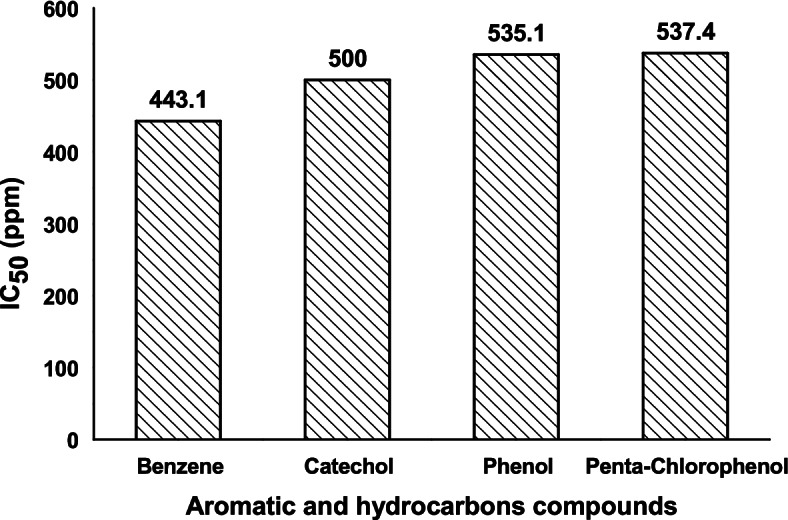
IC_50_ of aromatic and hydrocarbon compounds on the bioluminescence of *Vibrio* sp. 6HFE

The bioluminescence inhibition pattern of *Vibrio* sp. 6HFE in the response to hydrocarbons (benzene, catechol, phenol, and penta-chlorophenol) at concentrations (1 to 1000 ppm) was evaluated for 5 min. The IC_50_ values of them are demonstrated in Fig. 10. The bioluminescence of *Vibrio* sp. 6HFE was inhibited by benzene, catechol, phenol, and penta-chlorophenol at 443.1, 500, 535.1, and 537.4 ppm. Therefore, the toxicity order of them was arranged from the most toxic to the least toxic as the following: benzene > catechol > phenol > penta-chlorophenol.

- BIA for polluted samples﻿

**Fig. 11:**
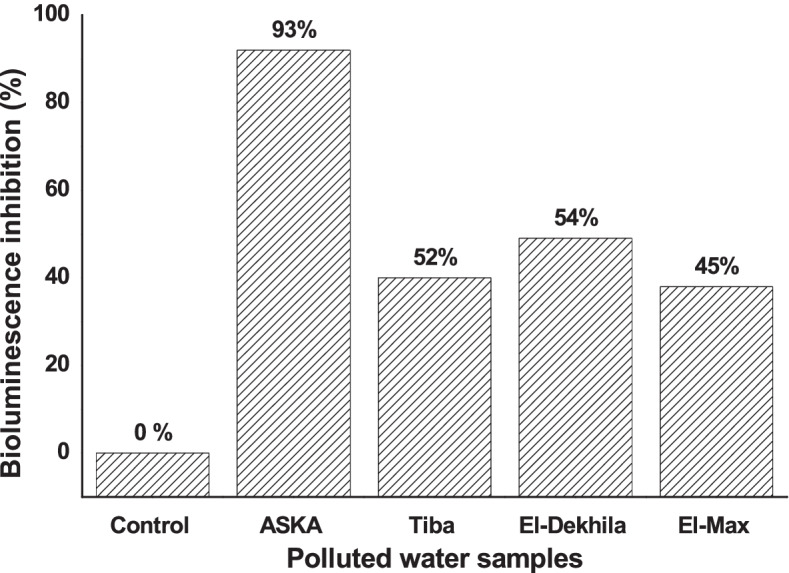
Effect of different polluted water samples on the bioluminescence of the biosensor *Vibrio* sp. 6HFE

The biosensor *V.* sp. 6HFE was employed for pollution detection in four different real ecosystems: two samples from industrial effluents and two others from polluted seawater (El-Dekheila and El-Max). The following Fig. 11 illustrates the bioluminescence inhibition (%) as a result of the exposure of *V*. sp. 6HFE to real polluted samples. As noticed, the effluent of AKSA Company resulted in 93% of bioluminescence inhibition implying a higher pollution percentage. However, the bioluminescence inhibition was displayed by 54%, 52%, and 45% for El-Dekheila’s seawater, Tiba’s Company, and El-Max seawater, respectively. Notably, the chemical analysis of examined samples revealed higher pollution with various heavy metals where AKSA contained Zn^2+^ ≈ 2.4 ppm and Fe^2+^ ≈ 0.54, Dekhila sample recorded Cr^6+^ 0.51 ppm and Fe^2+^ 0.39 ppm, and Tiba and El-Max samples had 0.6 and 0.38 ppm of Fe^2+^. However, Environmental Pollution and Legislative Regulations (Law 48. 1982 & Decree 8, 1993) of Egypt stated that the allowable limit of Zn^2+^ and Cr^6+^ must not exceed 1.0 and 0.01-0.05 ppm, respectively.

## Discussion

Several studies reported light emission from *V. fischeri*, *V. harvei*, *V. parahaemolyticus*, *V. alginolyricus*, and *V. vulnificus* [31, 32]. Many literatures assured the symbiotic relationship between the common cuttlefish (*Sepia officinails*) and the bioluminescent *Vibrio* species [33–35]. The isolated bacterial strain from *Sepia* belongs to the genus *Vibrio* and deposited in GenBank as *Vibrio* sp. 6HFE. It is observed that the bioluminescence of *Vibrio* sp. 6HFE was optimum at 25°C. Similarly, *V*. *fischeri* was able to grow and efficiently illuminated at a temperature range 20-26°C [36, 37]. Notably, the optimum temperature for luciferase was 25°C. As luciferase is temperature dependent, the bioluminescence exhibited higher intensity at the range of 25 to 30°C and fluctuated notoriously above 30°C as mentioned by [38–40].

Also, it is noted that the bioluminescence stability of *Vibrio* sp. 6HFE was raised by aeration. Such result could be explained by prober and homogenous distribution of media component and oxygen under shaking condition, where, under appropriate aeration conditions, the expression of lux operon enhances which subsequently elevates quorum sensing. Additionally, the agitation reduces the effect of exhaust gas produced during the incubation period [39, 41]. The relationship between the increasing of bioluminescence intensity and the growth curve of *Vibrio* sp. 6HFE revealed that the stationary phase has the highest bioluminescence intensity combined with stability. In agreement with these results, Thorn et al. [42] noted that luminous *Pseudomonas aeruginosa* MCS5-lite emitted stable bioluminescence during the stationary phase. Eventually, the process of bioluminescence is time dependent which relies mostly on bacterial cell density and subsequent autoinducer accumulation [43].

Out of statistical experiment results, yeast extract had a high effect of the bioluminescence intensity at low concentrations. This could be attributed to the light-blocking effect of yeast extract upon higher concentrations, where the extremely nutritive nature of yeast extract resulted in a much higher bacterial growth rate which ultimately led to much higher cell density that is proportional to the bioluminescence as previously reported by Lee et al. and Hassan and Oh [44, 45]. Otherwise, Ca^2+^and Mg^2+^ enhanced the bioluminescence of *V.* sp. 6HFE upon higher concentrations. Both cations act as cofactors for luciferase enzyme and in some species cofactors for photoprotein which appears in the absence of luciferase enzyme. Such chemical molecule is a combination of luciferin protein and oxygen which requires Ca^2+^ or Mg^2+^ for emitting light [46]. However, K^+^ improved the bioluminescence of *V.* sp. 6HFE, where it plays a special role in increasing the long-chain aldehydes and intracellular luminescent proteins and subsequently elevates the light output. Furthermore, it regulates the osmolarity process through affecting on the energetics of the outer cytoplasmic membrane and consequently the intensity of bioluminescence, which is affected by changing the cell respiration rate [47, 48]. Besides that, K^+^ in the medium increased membrane phosphorylation and transmembrane electrochemical gradient; as a result, it stimulated more bioluminescence at acidic condition [45, 47].

The process of light emission is somehow a reflection to the bacterial metabolic and enzymatic intensity and any inhibition occurred which led to a noticeable change in the luminescence production. Therefore, it is possible to utilize such property in monitoring and estimating the toxicity of any pollutant, even at low concentrations. Generally, the bioluminescence inhibition assay (BIA) becomes one of the most popular bioassays for the evaluation of toxicity in aqueous solutions [5, 6]. Accordingly, the bioluminescent strain *Vibrio* sp. 6HFE would be applied as a biosensor for detecting pollution. The toxicity of pollutant was determined in the form of IC_50_ within a short time (5-30 min) through the BIA approach as revealed by Cukurluoglu and Muezzinoglu and Halmi et al. [19, 20]. Broadly, such monitoring bioassay considers being a simple and fast detection approach by producing an alarm in the form of light on/off. In addition, it is a clean and cost-effective method in comparison to chemical methods [49, 50]. Out of the results, it is observed that *Vibrio* sp. 6HFE was found to be characteristic by exhibiting lower IC_50_ with the tested solvents compared to other studies [51]. It is found that just 2.0 ppm of zinc and cupper led to moderate inhibition of the bioluminescence intensity at only 5 min compared to *Photobacterium* sp. strains MIE and LuB-1 which recorded 0.85 and 1.08 ppm as IC_50_ for zinc and cupper at 15 min, correspondingly as stated by Halmi et al. and Hong et al. [15, 52]. In comparison to the results of other literatures [53], it could be concluded the efficiency of *Vibrio* sp. 6HFE as a promising biosensor for detecting various pollutants with considerable concentrations in wastewater. Therefore, the strain *V*. sp. 6HFE seemed to be advantageous in providing fast and immediate indication about the pollution burden in examined samples and give alarm for movement to rapid solution.

## Conclusion

The present study demonstrated the isolation, identification, and characterization of luminous bacteria from Abo-qir bay, Alexandria, for the first time. The selected strain *V.* sp. 6HFE exhibited the highest bioluminescence intensity as assessed quantitatively. The cultural conditions were optimized to maximize light intensity via OVAT, PBD, and CCD. By applying optimized conditions, the luminous *V*. sp. 6HFE was employed to detect the toxicity of various toxicants, including solvents, heavy metals, and hydrocarbons. The strain *V.* sp. 6HFE confirmed its efficiency as a biosensor for monitoring pollution in environmental real ecosystems.

## Data Availability

All data generated or analyzed during this study are included in this published article.

## Abbreviations

BL: Bioluminescence
LA: Luminescence agar
CPS: Count per second
PBD: Plackett-Burman design
CCD: Central composite design
BIA: Bioluminescence inhibition assay
OVAT: One variable at a time
